# Cultivating Skillful Means of Care in Schools Through Compassion Practice and Individual and Joint Inquiry

**DOI:** 10.1007/s12671-022-01867-x

**Published:** 2022-04-15

**Authors:** Ashley Seidel Potvin, William R. Penuel, Sona Dimidjian, Thupten Jinpa

**Affiliations:** 1 University of Colorado Boulder, Boulder, USA; 2 McGill University, Montreal, Canada

**Keywords:** Compassion, Care, Educators, Schools, Co-design, Joint inquiry

## Abstract

**Objectives:**

We examined the extent to which compassion practices helped guide skillful means of care among educators. We engaged educators in a collaborative design (co-design) process that foregrounded two components: (1) contemplative practice and (2) developing skill in how social interactions are embedded within wider systems through individual and joint inquiry. We analyzed the ways educators developed awareness of social suffering and set intentions to alleviate suffering. We examined how co-design fostered an understanding of compassion and new ideas about how to respond skillfully to suffering in schools.

**Methods:**

Using qualitative methods, we analyzed data from educators who participated in co-design, including their written reflections, field notes, semi-structured interviews, and surveys.

**Results:**

Educators identified multiple opportunities for acting with compassion, including approaching school-based interactions with compassion, cultivating compassion for themselves, and envisioning school change through a lens of compassion. Educators’ experiences in co-design directly informed how they imagined compassionate action in their schools. Specific elements highlighted were contemplative practice, reflection, and individual and joint inquiry.

**Conclusions:**

The adaptation of a general program on compassion training can benefit from attending to how to show compassion in the context of concrete interactions in schools, and this can support educators in developing skillful means of care. Our analyses provide insight into the components that supported educators to offer compassion and suggest that educators’ skillful means of care can be cultivated through both contemplative practice and inquiry into social suffering. We offer a conceptual model for developing skillful means of care educational settings.

While schools can be sites of joy, they can also be sites of suffering for students and teachers alike (Dumas, 2014; Garcia, 2019). All too often, policies and practices, such as grading, discipline, and accountability can cause pain and harm (Mallett, 2017; Marion & Shepard, 2021; Scott et al., 2017; Wiley, 2021). School structures that rely on sorting, labeling, and tracking students can leave students feeling ostracized, different, or less than (Oakes, 2005). For educators, policies imposed on them by others (Kelchtermans, 2005) and conflict among colleagues (Achinstein, 2002) also can be sources of suffering. In addition, enduring challenges for educators, such as burnout, stress, isolation, overwhelm, and fatigue from experiencing and witnessing trauma and pain, has been felt more acutely during the COVID-19 pandemic (CDC Foundation 2021; Diliberti et al., 2021; Herman et al., 2018). In this context, educators have been forced to navigate their own grief and loss while also supporting the social and emotional needs of their students, oftentimes with little support (Ewing, 2021; Minke, 2020). Moreover, systemic racism and oppression that occur in larger society, such as ongoing injustices and state-sanctioned violence against Black, Indigenous, People of Color (BIPOC), are often replicated and, at times, amplified in schools, making schools into “sites of suffering” for members of those communities (Dumas, 2014). While these practices, policies, and routines can cause harm to all members of school communities, the consequences are experienced most acutely by members of non-dominant communities, including BIPOC and lesbian, gay, bisexual, transgender, and queer and questioning (LGBTQ) students and educators. Responding to suffering in schools requires attending to interpersonal and intrapersonal skills and to the systemic factors that perpetuate suffering.

Educators can attend to suffering in schools through the development of skillful means of care. One way to develop such care is through contemplative-based training in compassion, an area that has recently begun to be explored in education (Jennings et al., 2017; Roeser et al., 2013; Taylor et al., 2016). Another is in developing awareness and skill in social interactions, as well as in how social interactions are embedded within wider systems (Garza, 2009; Valenzuela, 1999). We focus on a program for educators that pursues in concert contemplative-based training in compassion as well as the development of awareness and skill in how social interactions are embedded within broader systems. Using the encompassing lens of care for two ideas from Buddhist psychology, one that refers to a practice for cultivating compassion (*karuna*) and its skillful application in interaction (*upaya-kaushalya*), we sought to bring these two supportive conditions for development into a single program to support educators in responding to the specific forms of suffering in schools. We define compassion as “a sense of concern that arises when we are confronted with another’s suffering and feel motivated to see that suffering relieved” (Jinpa, 2015, p. xxii). Simply stated, while cultivating compassion focuses on our intention, attitude, and feeling of connection in the context of an interaction, “skillful means” pertains to how one might translate those aspects of compassion into action that is constructive and beneficial to others and that creates conditions for care to develop more widely in specific communities and organizations. Thus, “skillful means of care” unites two key concepts, compassion and skillful means, to include both the awareness or discernment of human need and the situation to act in ways beneficial to others.

Schools are complex systems, and a wide range of studies points to both the challenges and benefits of cultivating caring relationships between teachers and students, between school staff and families, and among colleagues (e.g., Antrop-González & De Jesús, 2006; Garza, 2009; Roberts, 2010). For educators to learn to care skillfully—that is, to attend to individuals’ needs—they need an understanding of their own and students’ situations. Students from every cultural community bring particular conceptions of care and preferred ways of being cared for by others, and these can differ for students from different communities. In a study comparing Latinx and White students’ perception of teacher care, Garza (2009) found differences between a sample of Latinx and White students’ perceptions of caring behaviors of students, in terms of the priorities they gave to the importance of different expressions of care. A related study focused on African American students’ perceptions of teacher relationships found that teachers showed care by helping students navigate an inequitable system (Roberts, 2010). And while studies often find some common characteristics of what makes a teacher caring to students (e.g., kindness, flexibility), studies that attend to difference tend to find some distinctive characteristics of caring teachers for students from particular non-dominant communities (e.g., Antrop-González & De Jesús, 2006; Dexter et al., 2016). These studies point to ways that cultural norms, structural inequalities, and systemic racism all shape both educators’ and students’ responses to suffering, making it challenging for educators to skillfully care for their students (Conchas & Vigil, 2010). Other studies point to how the work of caring itself is highly gendered, and often is invisible, undervalued, but expected labor of women educators within schools (Acker, 1995; Collins, 2000; Puig de la Bellacasa, 2017; Warin & Gannerud, 2014). Identifying the components of skillful means of care makes visible the largely invisible labor of care in schools and will require a shift in the responsibility to attend to suffering in schools to all members of the school community. Considering the multiple, intersecting inequalities of student and adult relationships in schools, then, what it means to develop skillful means in care presents challenges to intervention designers, not only in terms of how to support the development of a practice of compassion, but also in supporting educators in noticing and responding to the forms of social suffering that arise in schools.

To support educators’ development in the skillful means for care to respond to the suffering in their schools, we engaged educators in collaborative design, or co-design, of a digital course on teacher leadership in compassion and dignity for other educators. Our aim was to design a course that supported educators in cultivating compassion for self and others, in recognizing the dignity, or essential worth and value of self and others, and in bringing such knowledge and skill into their schools as leaders. Co-design is a humanizing approach to research with educators (Potvin et al., 2021) that attends explicitly and intentionally to relationships and context (Penuel et al., 2007; Potvin, 2021). Co-design as an approach to research and design is “a highly facilitated, team-based process in which teachers, researchers and developers work together in defined roles to design an educational innovation, realize the design in one or more prototypes, and evaluate each prototype’s significance for addressing a concrete educational need” (Penuel et al., 2007, p. 53). The co-design process foregrounds team members’ lived experiences and contexts, and it is through this process that we explored the complexity of schools as systems, the application of compassion in schools, and educators’ responses to the forms of suffering specific to schools. For these reasons, co-design is an apt approach to research and design of a digital course focused on compassion, dignity, and leadership for educators. To support educators’ development of skillful means of care, the co-design process foregrounded two key components: (1) contemplative practice and (2) developing awareness and skill in how social interactions are embedded within wider systems through individual and joint inquiry. The current study’s co-design process was unique as it provided opportunities for educators and researchers to engage in both contemplative compassion practices and joint inquiry to study and discuss compassionate interactions in schools and their responses to them.

The Center for Contemplative Mind in Society (n.d.) defines contemplative practices as those that “cultivate a critical, first-person focus, sometimes with direct experience as the object, while at other times concentrating on complex ideas or situations.” Contemplative practices encompass a wide variety of practices from global traditions. In this paper, we refer specifically to contemplative practices embedded within Compassion Cultivation Training (CCT): settling the mind, intention setting, compassion and kindness for a loved one, compassion and kindness for oneself, embracing common humanity, cultivating compassion for others, and active compassion. As part of the co-design work, a group of teachers, counselors, and school administrators first participated in contemplative compassion practices through the 8-week CCT. This same team then continued to engage in compassion practices together throughout the ongoing co-design process.

One area of research on compassion in schools for educators has focused on providing educators with skills and resources for addressing their own wellbeing and social and emotional competencies so that educators can be better prepared to support student wellbeing, student outcomes, and school and classroom climate (e.g., Jennings & Greenberg, 2009; Meiklejohn et al., 2012; Roeser et al., 2012). These interventions typically involve training in mindfulness practices and may also incorporate compassion and kindness practices as components of a larger program for teachers (e.g., Braun et al., 2020; Jennings et al., 2017; Roeser et al., 2013; Schussler et al., 2016; Taylor et al., 2016). A small set of studies has focused on the effects of explicit compassion training for educators and has found a number of benefits to compassion training. These studies have found that teachers reported developing adaptive strategies for coping with stress, increases in practicing kindness and compassion, a tendency to view students whom they typically experience as challenging more positively, and improvements in their ability to forgive colleagues and students (Braun et al., 2020; Taylor et al., 2016). A study of a program focused on improving the wellbeing of educators found that educators who engaged in compassion practices reported increases in self-compassion and decreases in self-criticism and reported applying compassion techniques to their interactions with colleagues and students (Maratos et al., 2019).

These studies build on work in healthcare and with the general population that demonstrate the impact of training in compassion meditation on prosocial behavior (Ashar et al., 2016; Brito-Pons et al., 2018; Condon et al., 2013; Jazaieri et al., 2015; Leiberg et al., 2011; Raab, 2014; Sinclair et al., 2021; Weng et al., 2013), one of which shows that increases in helping behavior were related to the amount of time people spent in engaging in meditation practice (Leiberg et al., 2011). One mechanism by which compassion meditation achieves this effect may be the observed effect of meditation practice on people’s emotional responses to the suffering of others. A longitudinal, neuroscientific study of compassion training showed that meditation enhanced positive emotional responses to the suffering of others (Klimecki et al., 2013). Other studies have demonstrated that compassion training has the potential to buffer against empathic distress when faced with suffering (Ashar et al., 2016). Research also highlights the benefits of training for increasing self-compassion and cultivating a more open, curious relationship toward one’s own experiences of difficulty with others (Neff & Germer, 2013). Based on this work, we posit that contemplative practice is an important vehicle for supporting the development of skillful care.

In addition to contemplative compassion practice, the second key component of the co-design work with educators was that of individual and joint inquiry in developing skillful means of care. Skillful means of care in schools requires noticing suffering in others, as well as particular ways of interpreting suffering (Worline & Dutton, 2017). Much suffering at school can be invisible, and it can manifest as people not meeting expectations; therefore, effort may be needed to begin to notice suffering’s presence and become curious about it. Similarly, educators may benefit from learning to discuss suffering that is noticed with co-workers, since such discussion of suffering can activate compassionate responses within an organization (Dutton et al., 2006). However, simply noticing suffering is not sufficient for skillful means of care, since people’s response to suffering in the workplace is shaped by their appraisals of suffering (Atkins & Parker, 2012; Rynes et al., 2012). Judging someone to be undeserving of compassion or as deserving suffering as a consequence of their actions can lead people to turn away from suffering; conversely, cultivating a more generous, curious attitude toward manifestations of suffering can lead more readily to compassionate action (Worline & Dutton, 2017). Moreover, the tendency to interpret responses to suffering in a way that opens to compassion is often prejudiced by one’s own positionality—within families and culture, organizations, and encounters with stereotypes perpetuated by different social systems (Goetz et al., 2010). Seeing people as having inherent dignity and worth and cultivating the ability to be present to others’ suffering can facilitate compassionate responses to suffering and support skillful means of care (Worline & Dutton, 2017).

We sought to support educators in developing skillful means of care through the practice of field noting as part of the individual and joint inquiry process. The practice of educators writing field notes to notice their micro-interactions is relevant to compassion because field noting can serve as a vehicle for supporting educators to see suffering in schools that they are not likely to otherwise see. Field notes are detailed accounts of interactions that seek to distinguish low-inference observations and reflections of individuals on those observations (Emerson et al., 1995). They provide an opportunity for educators to reflect—after the fact—on their interpretations of their own and others’ actions in ways that can help them distinguish the inferences they make from others’ motivations or intentions from their observations.

Developing field notes does not help educators to escape bias in what they notice or replace judgments of others with more generous interpretations (Emerson et al., 1995); here, there is a critical role for theory in helping educators interpret their actions differently (Cole & the Distributed Literacy Consortium, 2006; Jurow et al., 2012). Guidance from theory often seeks to counteract educators’ less than generous interpretations of others’ intentions and motivations, particularly when they reveal deficit perspectives of students and their families (Van Steenis, 2021). Thus, a key feature of these arrangements is joint inquiry into field notes. Such inquiry further helps educators to develop perspective on their own noticings and engage with alternative interpretations of interactions (Creese et al., 2008), and being able to look across sites generates perspectives on how the particularities of context and history matter (Vossoughi & Gutiérrez, 2014). In the co-design process, educators had the opportunity to share and discuss their field notes with other educators. In addition to supporting educators’ noticing, the field notes were intended to help develop content for the course, by identifying specific situations where skillful care might be applied to alleviate suffering in schools.

Through our work with educators in the co-design process, we became curious about the ways in which educators applied compassion in their school contexts to address the social suffering they witnessed and/or experienced. We wondered how educators demonstrated skillful means of care, that is, how they offered compassion skillfully through awareness and discernment of others’ needs and the situations. Educators’ skillful means of care within a school context could take on many forms, including attuning to students’ suffering and generating ways to alleviate the suffering in their classrooms and schools as well as attending to suffering while still preserving their own self-care.

We examined the extent to which compassion practices helped to guide skillful means of care among educators in schools. Specifically, we explored how educators in the co-design team reported offering compassion in their schools. We analyzed the ways educators related with care to their own thoughts and emotions and to the suffering of others, developed awareness of the sources of social suffering in schools, and set intentions to alleviate those forms of suffering. We also examined the ways in which the experience of co-design fostered not only an understanding of compassion but also new ideas about how to respond skillfully to suffering in schools. To understand the ways in which educators demonstrated skillful means of care for themselves, for students, students’ families, and colleagues, and for their schools, we asked, specifically: (a) How did educators approach social interactions in their schools through a lens of compassion? (b) What aspects of the co-design process informed how educators think about compassionate schools?

## Method

### Participants

Educators were invited to participate in the co-design process via an email written by researchers and sent to educators in one school district by a district administrator on behalf of the research team. The school district is located in the Rocky Mountain Region in the USA and serves approximately 29,000 students from PreK-12. Within the district, 67% of students identify as White, 19% identify as Latinx, 6% identify as Asian, 6% identify as two or more races, and 1% identify as Black. Approximately 20% of students receive free or reduced lunch, and 9% are multilingual learners. The research team had worked with this district previously and had established a partnership.

Educators interested in participating in the co-design process completed a brief application on which they were asked to provide information about their position, grade level(s) and content area(s), and school as well as reasons for their interest in the project and prior experience with compassion, leadership, professional development, and curriculum development. Twenty educators applied for the co-design process, and 16 were selected to participate because of the diversity of roles they held within the district (e.g., counselor, teacher, administrator), their stated interest in the project, and their experience with the relevant topics. Due to scheduling conflicts, six educators chose not to participate in co-design. The ten educators who participated in co-design were eligible and invited to participate in this study, and 100% of educators who participated in co-design consented to participate in the study in accordance with the university’s institutional review board procedures. Due to the qualitative nature of this study, we did not conduct a power analysis.

Participants in the research study included five teachers, three counselors, one librarian, and one principal, and they represented six different elementary and middle schools in the district. Seven educators worked in middle schools, and three educators worked in elementary schools. Ninety percent of educators self-identified as White (*n* = 9) and 10% as White and Latinx (*n* = 1). Eighty percent of educators identified as female (*n* = 8), and 20% of educators identified as male (*n* = 2). Educators had between 4 and 30 years of experience working in schools, with an average of 15 years.

### Procedure

The ten educators collaborated with six researchers to design a digital course for educators that explores the ways in which practices of compassion and a focus on the essential dignity of educators and students can contribute to the wellness of educators and schools. Co-design meetings occurred weekly for 2 hours from October 2019 through May 2020, for a total of 58 hours. The team of educators and researchers began the collaboration by completing CCT together. CCT was developed by the fourth author, in collaboration with a multidisciplinary team at Stanford University. CCT is an 8-week compassion training focused on the growing science of compassion and secular practices for cultivating compassion adapted from Tibetan Buddhist practices (Goldin & Jazaieri, 2017). Through CCT, participants cultivate compassion, kindness, and empathy. CCT is taught by certified facilitators and follows a consistent structure. Each 2-hour session integrates interactive discussions, lectures, meditation practices, real-world exercises, and inquiry. Participants are asked to commit to a daily home practice that includes a 20–30-minute compassion meditation as well as informal practices. CCT is structured around 6 steps that participants are introduced to incrementally throughout the 8 weeks: settling the mind through breath awareness, compassion and kindness for a loved one, compassion and kindness for oneself, embracing common humanity, cultivating compassion for others, and active compassion. Participants are also introduced to intention setting as a way to connect to deeper values and aspirations.

Following the 8-week compassion training, the team met weekly to adapt, extend, and apply core content, practices, and activities from CCT in the design of a compassion course for educators. The team met in person until March 2020, when the COVID-19 pandemic resulted in statewide stay-at-home orders; at that point, meetings moved to a virtual platform until May 2020. Three of the university researchers on the co-design team, the first three authors, served as facilitators. Together, facilitators planned the weekly meetings, developed facilitator guides, and led the meetings. Meetings focused on engaging in compassion practices to support wellness, analyzing sources of suffering in schools, and designing content for the compassion course. Meetings began and ended with a guided meditation practice for cultivating compassion and often included opportunities to debrief the practices and to check-in with one another. The middle of the meetings focused on design work, and participants were encouraged to draw upon their lived experiences, both inside and outside of school, to ensure the designed course was relevant and accessible for educators. Facilitators situated design work within educators’ school-based contexts and introduced readings and frameworks that sought to put individual student’s suffering in a social and historical context (e.g., Dutton et al., 2006; Garza, 2009; Ginwright, 2018).

Throughout the design process, participants engaged in reflection and joint inquiry. A subgroup of participants volunteered to write field notes about their experiences with compassion in schools and shared and discussed them with other participants in the subgroup. The team reviewed the field notes, the author shared reflections about the process of writing field notes, and the group discussed the field notes following a discussion protocol. The field notes discussion protocol included prompts for the field note author, such as “What was the process of writing field notes like? How did you choose this moment to focus on?” The discussion protocol also included prompts for the readers, such as “How did you feel as you read? What is there to celebrate about this field note?” Finally, the discussion protocol included prompts for everyone in the group to discuss, including “What connections to compassion do you see?” and “Was it difficult to stay low inference in your observations? And if yes, why do you think this was so?” Writing and sharing field notes supported educators to cultivate compassion and identify opportunities for compassion in their school communities and served as fodder for the design of the course.

During the co-design, we collected data using the measures below to answer the research questions for the current study. From October 2019 to December 2020, all participants responded to a series of brief written reflection prompts directly following each of the CCT sessions. We audio recorded 13 co-design meetings from February 2020 to May 2020. From March 2020 to May 2020, a subgroup of six educators volunteered to record one set of field notes as part of the co-design process and these field notes were also collected as data for the study. The other four educators opted not to write field notes because of the additional time commitment it required beyond the regular co-design meetings. At the conclusion of the co-design process, we interviewed eight educators. In January 2021, we sent a brief follow-up survey to all participants and six responded.

### Measures

#### Written Reflections

Written reflections were collected and analyzed to identify the ways in which educators reflected on the topics and components of compassion training reported on offering compassion in their school contexts.

#### Field Notes

The six educators who volunteered to record one set of field notes each used a template to identify the situations that were relevant for compassion in schools and how they thought about offering compassion to themselves and/or others in these situations. Educators identified a 10–15-minute interaction they had or witnessed in school that demonstrated compassion or provided an opportunity for compassion.

#### Semi-structured Interviews

A member of the research team who participated in the co-design intervention but did not facilitate the meetings interviewed eight educators at the conclusion of the co-design project using a semi-structured interview protocol to identify the ways that educators offered compassion in their school contexts.

#### Survey

Eight months after the conclusion of the co-design project, six educators completed a brief follow-up survey with six open-ended questions, asking about their perceptions of skillful and unskillful application of compassion, for specific examples of skillful acts of compassion, and the ways in which they offered compassion in their school leadership and during the pandemic.

### Data Analyses

Skillful means of care requires contextual specificity, and so we used a sociocultural approach to analyzing interactions to help us analyze the data and understand how educators offered compassion in their contexts. We began our analysis with our first research question: How did educators approach social interactions in their schools through a lens of compassion? We used inductive and deductive coding to analyze the data (Miles et al., 2014). We engaged in first-cycle coding, beginning by analyzing educators’ written reflections to look for the situations they identified as opportunities for compassion and the ways they distinguished between skillful and unskillful responses. Transcripts from the 13 co-design meetings were used to corroborate key findings that emerged from the field notes, reflections, interviews, and surveys.

We then engaged in second-cycle coding guided by Rogoff’s (1995) three planes of analysis to consolidate codes and identify salient themes. This framework affords us the ability to foreground interactions that take place on each plane, one at a time, while using the other planes as background information. We used the interpersonal plane of analysis to focus on how educators approached their interactions with school community members with compassion. We used the personal plane of analysis as a lens for identifying themes related to the ways in which educators offered compassion to themselves. We used the community/institutional plane to focus on how educators envisioned school community change through a lens of compassion. This step of second-cycle coding allowed us to see emergent patterns related to each of the three categories.

In the process of identifying the emergent patterns, we became curious about what motivated educators to offer compassion across these three planes. We engaged in one more cycle of coding to look for what educators attributed to their patterns of interactions and thinking about compassionate schools to address our second research question: What aspects of the co-design process informed how educators think about compassionate schools? The first author wrote analytic memos, summarizing emergent themes (Miles et al., 2014). One memo, for instance, summarized opportunities for compassion in schools while another memo summarized the codes that emerged from the first-cycle coding process. Memos were shared with the author team. The author team reviewed the codes and memos, noting that similar codes emerged across all data sources. Key themes were discussed among the author team and checked against the data corpus.

## Results

Results demonstrated that educators identified multiple opportunities for offering compassion, including approaching their school-based interactions with compassion, cultivating compassion for themselves, and envisioning school change through a lens of compassion. We provide an example of each theme; examples were carefully selected as representative of the theme within the data corpus. It was also important to us, given the participatory nature of co-design, to ensure that a variety of educators’ perspectives were included in this study as opposed to focusing on just one or two educators’ experiences through the selected examples. Additionally, results demonstrated that the experience of educators in co-design directly informed how educators imagined compassionate action in their schools; specific elements highlighted by educators were contemplative practice, joint inquiry, and reflection, and they also pointed to the importance of the interaction of all three elements.

### Interpersonal: Educators Approach School-Based Interactions with Compassion

Educators most often identified interactions with school community members as opportunities for demonstrating compassion in their school contexts. Interactions with students, interactions with colleagues, and interactions with students’ families emerged as themes in the data.

#### Interactions with Students

The most frequent theme that emerged related to educators’ opportunities to offer compassion during interactions with students. Common situations identified by educators included managing a classroom and building classroom community, identified by all ten educators, and supporting students experiencing difficulties in their home lives, identified by nine educators.

Approaching classroom management with compassion emerged as a major theme within the data and appeared across all data sources (reflections, educator reflections, surveys, interviews). Educators reflected that classroom management was a professional challenge they experienced that related to compassion. Approaching the interactions from a place of compassion helped educators to gain insight and discernment. Offering compassion to their students supported educators in letting go of the need to control the situation or students’ behaviors, and it freed educators up to see how students might be suffering and to find new ways to approach their students. Donna (all names are pseudonyms), for example, chose to write her field notes about a challenging interaction with a student during her third-grade library lesson. Donna moved several students’ seats because they were talking during the lesson, and one student, Sarah, became angry, pushing a chair and declaring “I hate being here.” Later in the class period, Donna asked Sarah why she had moved her seat, but Sarah refused to talk. Donna became aware that the student might be suffering and decided to follow up with her the next morning, inviting Sarah to sit with her on the floor of the hallway. Donna listened and Sarah spoke about why she was so upset. Donna gained a new perspective on her experience and recognized her suffering. Donna reflected that she wished “instead of asking her why I had moved her, trying to get her right back on task … I had asked her what she was feeling” (Donna’s Field Notes). Donna also reflected that this situation “showed that making the connection did something, the added connection, but it’s going to be very much an ongoing process” (Transcript_200303). In this example, Donna recognized her initial desire to control the student’s behavior and the situation during class and the ways in which this stance overtook her capacity to attune to her student’s suffering in the moment. Later, Donna discerned that Sarah might be suffering in some way, and she returned to Sarah and the situation the next day with more openness. Donna, like other educators in the study, applied compassion in her interactions with students and noted improved interactions and relationships with them as a result.

After sharing her field notes with the group, Donna revealed that she hesitated to share this example because it was so challenging but that the “reason I chose it was because it was difficult, because there were things I wished I had done differently in hindsight” (Meeting transcript_3.3.20). Donna shared that the situation with the student was made additionally challenging by the pressure she felt to adhere to a school-wide mandatory classroom management curriculum. She described the classroom management curriculum as “very scripted” and that it “doesn’t really mesh with what I see as being a compassionate educator.” Mia responded,

I felt definite common humanity of having these moments that are frustrating, that are difficult and that when you get a chance to step away from it, you rethink it … The celebration is like, what a gift to be able to actually have that pause where you can reflect and to say, “Okay, I’m going to step back in and let’s try this again, or let me just frame this in a different way, even in relationships.” (Meeting transcript_3.3.20)

Siena also pointed out that Donna “gave these two other opportunities to practice [compassion with the student]” and that “I think it’s a practice of [common] humanity that you show we’re all connected and then the compassion grows out of that” (Meeting transcript_3.3.20). In this example of joint inquiry, Mia and Siena responded to Donna’s sharing with tenderness and identified common humanity (a core element of contemplative compassion practice and CCT) as an important feature embedded in Donna’s field notes, reflection, and the group discussion. Mia noted feeling a sense of common humanity with Donna in that moment and Sienna highlighted Donna’s ability to see common humanity with her student when she followed up with her in the hallway the next day.

#### Interactions with Colleagues

Educators also identified interactions with colleagues at school as opportunities to extend compassion. In written reflections, interviews, and field notes, educators shared struggles related to colleagues whom they experience as challenging as well as a desire to bring compassion to their interactions. For instance, one educator disagreed with the ways in which her coworkers approached students and addressed students’ behaviors. Ruth shared, “At work I am faced with the challenge of being compassionate toward colleagues who operate very differently than I do, who are not always kind in their communication, and who I’d prefer to avoid if given the chance” (Ruth Reflection). Regardless of why these interactions were challenging, educators brought awareness to them and to their emotional responses, noting that they could be “impatient” with colleagues. Martin shared, “The practice of seeing the shared humanity in all of us seems very useful, especially when dealing with difficult people. Also, extending compassion to all without ‘liking’ everyone is a great practice for my job” (Martin Reflection). Through the compassion training, educators recognized that all people, including colleagues with whom they disagreed, were deserving of compassion.

It was not just challenges with colleagues that educators viewed as opportunities to offer compassion, but also in being moved by witnessing their colleagues’ suffering. Jessica, for example, noticed that her colleagues were “very worn out after years of struggling with the administration” and that their suffering was only exacerbated during the global health pandemic. Seeing their suffering moved Jessica to advocate for her colleagues, explaining,

I continue to take up the role of mouthpiece. I see the staff suffering, unable to work together, not able to enjoy their work much of the time, so I constantly fight for them even when it isn’t my fight. (Survey)

For Jessica, and for several other educators in the sample, seeing their colleagues suffer motivated them to act with compassion. This example also highlights that educators, such as Jessica, viewed skillful application of compassion as linked to advocacy.

#### Interactions with Students’ Families

Educators also identified their interactions with students’ families as opportunities for care and compassion. Most frequently, educators spoke or wrote about offering compassion during meetings with students’ families related to their child’s progress. Bridgette, for example, used intention setting that she learned from the compassion training before meetings she anticipated would be contentious. Prior to calling a parent to talk about her daughter’s behavior in class, she set an intention to have compassion for the parent and to focus on their shared wish—that the student be happy and successful in the classroom. Bridgette described the start of the call, noting that the parent was “screaming” at her, upset with Bridgette’s teaching and that her child “wasn’t learning anything.” Bridgette breathed deeply as she listened to the parent voice her concerns. She explained, “I was thinking, I know that she just wants what’s best for her daughter and I tried to relate to her” (Interview). When the parent finished talking, Bridgette then responded calmly, explaining the situation and shining a light on the many successes the student had in class. The two spoke for a long time, and by the end of the call, the parent even thanked Bridgette. Reflecting on the situation later, Bridgette referred to this interaction as “the best phone call with the parent that I had” (Interview). Bridgette shared, “I was really proud of myself with the way I handled it. And I think it really had a lot to do [with] being able to discuss this stuff [compassion] in the [co-design] meetings and the compassion course” (Interview). Bridgette approached her day-to-day interactions with students’ families with tenderness, empathy, and compassion, and in ways that allowed her to recognize the dignity of her students and their families and to care for them. In this example, she set an intention, connected to their shared hopes for the student; used breath awareness to remain present, to listen, and to attune to the parent’s concerns; and engaged in a productive conversation about the student’s strengths and struggles. Like Bridgette, other educators in our sample spoke about drawing on their compassion practice to engage with families with care.

Educators also noted that moments of crises, such as the global health pandemic, were opportunities to extend compassion and care to students’ families. When school switched from in-person to remote learning due to statewide stay-at-home orders, Jessica maintained consistent and more frequent communication with her students’ families. She explained,

I talked to a ton of parents. They were sort of panicking and dealing with this, and kids were emailing me like, “I’m missing this assignment.” And so really being able to say, “You are okay. We are okay. This is okay. Let’s make sure we’re keeping everything in perspective.” I think that was just super helpful to everyone in all these situations. (Interview)

Jessica recognized the many challenges that arose during the pandemic, and that her students and their families were struggling and “panicking” during this time. She offered her presence through constant communication and offered reassurance. When Jessica said, “We are okay,” she positioned herself in solidarity with her students and their families, recognizing their interdependence and that they all suffered because of the pandemic. Jessica placed her care for students and their families over the academic demands and structures, “keeping everything in perspective.” Like Jessica, other educators in our sample viewed the global health pandemic as an opportunity to extend compassion to students’ parents/families.

### Personal: Educators Approach Their Internal Experiences with Compassion

Educators viewed their interactions as not just opportunities to approach their interactions with school members with compassion, but also as opportunities to cultivate compassion for themselves during or after these challenging exchanges. Educators spoke about offering themselves compassion related to their relationships with students, their colleagues, and students’ families. In addition, they described self-compassion as an important tool for their own wellbeing in the face of stress, self-criticism, and overwhelm they felt in the midst of a challenging school year.

Educators identified some of the more challenging interactions as opportunities to offer themselves compassion. As illustrated in the previous section, educators, for instance, spoke frequently about challenges related to interactions with students, including classroom management. A mention of classroom management in interviews and reflections was frequently accompanied by a recognition that educators needed to offer themselves compassion. In descriptions educators provided of challenges they experienced with students, they often reported feeling frustrated and ineffective as a teacher for a perceived lack of success in addressing these challenges. Recognizing that their acts of care for students were not always reciprocated as they would have liked or that they could not always control the outcome of their interactions was an act of self-compassion for educators. Bridgette explained, “Teachers must have self-compassion and let go of the fact that we can’t always control the outcome, even when we do our best to show compassion to students” (Reflection). Educators also noted that consistent challenges with students could leave them feeling exhausted or fatigued, and they recognized that compassion practice was a much needed tool for continuing to care for themselves and their students. Following one of the CCT sessions, Martin reflected on the potential of the loving-kindness practice:

I work with many suffering students and they can often be unpleasant or disruptive in class or simply energy draining because they have so many needs on an almost daily basis. I could really use instruction in cultivating “loving-kindness” for them and for myself since I very much struggle with compassion fatigue. (Martin Reflection)

Educators also viewed their interactions with colleagues as opportunities to offer themselves compassion. As described previously, several educators wrote or spoke about colleagues with whom they experienced difficulty. Rather than focus on changing their colleagues, these educators applied a compassionate lens to their interactions and reflected on the importance of offering themselves compassion. Ruth struggled in her interactions with a few colleagues and shared that “[I] am having to find compassion for myself given the discomfort I feel when talking with [these colleagues], when having to discuss issues with them” (Reflection). Ruth recognized that she could not avoid her colleagues, so she turned to self-compassion as a strategy for caring for herself during these challenging interactions.

Educators also reflected that interactions with students’ families presented yet another opportunity to bring compassion to themselves. As Martin explained,

I don’t know that we can change families and how they see teachers … But at least teachers can have that self-compassion, as far as doing the best that you can and understanding that you’re not always going to get the results you want or that you really visualized…So I think compassion on both sides. For teachers and staff as far as taking into account where our families, the students are coming from. And then cutting ourselves some slack. (Interview)

Educators found self-compassion to be a necessary and supportive practice during challenging interactions with students’ families.

Educators spoke and wrote about the stress they experienced working in schools and viewed self-compassion as a tool for helping them to navigate the stress, remain present with their students, and reconnect with the joys of teaching and leading. Educators shared that feelings of self-judgment and self-doubt were commonplace for them and increased feelings of stress and overwhelm. At the start of the study, Mia was “consumed by stress” at school and reflected that the co-design project and compassion training “couldn’t have come at a better time.” In early written reflections, Mia wrote about her “own suffering,” sharing that “stress fueled by self-critique has been raging through me these past months.” Mia found “the work around self-compassion to be life changing, deeply impactful to my work.” She admitted that in “November I was ready to quit [teaching].” She began to “cultivate a regular morning practice” that “shifted everything,” which she attributed to her learning in CCT and advice from a good friend. She explained, “the way I was showing up at school, the way my self-critical voice quieted and I was able to interact with that a little differently then that in turn impacts my students or colleagues or every, you know, all of us within that mix that dynamic” (Interview). Establishing a self-compassion routine for Mia “had a huge significance in nourishing my being and ramping down the stress” (Interview). Several other educators in the study also reported experiencing fatigue and burnout, and that self-compassion was “the first step to really dealing with that” (Bridgette Interview). When educators had the opportunity to engage in inquiry about these challenging experiences, such as through a written reflection, field notes, or a small group discussion within the co-design intervention, they observed specific means by which they brought self-compassion to the situation.

### Community: Educators Approach School Change Through a Lens of Compassion

One of the goals of the co-design project was to design a course that contributed to educators’ capacities to imagine and create more just and compassionate schools. We thus spent time in meetings engaging in inquiry and discussion about what it means to work toward school change. Educators identified specific means by which compassion could be applied in the context of envisioning school community change. Most frequently educators imagined changing school routines or policies through a lens of compassion. Educators identified grading and discipline policies as two school practices that caused pain and suffering to students and that could and should be revised. Viewing these policies through a lens of compassion was made all the more salient for educators during the global health pandemic, when remote learning spurred national conversations about equitable access to technology and schooling as well as how to support the mental health and wellbeing of students. Bridgette shared,

With this online situation we started talking about how we should grade them, and how there’s all these inequalities of these students, you know that have access or don’t have access to different things and have different life situations, and thinking about how that’s always the case. You know, this kind of just highlighted it with [the] online situation...It just kind of made me think of the overall system and how you know, it’s unequal and there’s so much value kind of placed on grades...I think that the system doesn’t always allow us to you know, have the space to have compassion for those students and help them to do their best. (Interview)

Bridgette, like several other educators in the group, began to see how caring for students was about more than just their individual interactions with students, with co-workers, or with families. Bridgette articulated the importance of being aware of structures and policies, and especially the way they create inequities for students in schools, as necessary for acting with compassion. Bridgette, like other educators in the group, recognized that many of the long-standing routines and policies within schools actually undermined compassion. As Jessica explained,

Compassion always seems to butt up against accountability...or things like compassionate educating kind of butts up against grading. And compassionate discipline kind of butts up against punitive measures. And so those I think will be the things that I want to really outline in terms of the things I am in control of, in terms of my classroom, and then things that I will want to be part of the conversation as a school, as a building, as a community. (Interview)

Jessica observed in her school that care and compassion did not underpin current policies such as accountability, grading, and discipline. Through learning about compassion practices, Jessica began to envision a school where policies were driven by care for students.

Some educators “got really excited” about the pivot to collective action and challenged other educators during co-design to accept responsibility for social and educational inequities by imagining and designing for more radically inclusive schools. By the end of the co-design project, Jessica, like other educators in the group, began to see opportunities where they could shift these policies through a lens of compassion. Educators brainstormed ideas to enact school change, such as creating working groups at their school to revise grading policies to reduce the pain associated with giving and receiving grades. Educators also began taking up practices of compassion to shift systemic structures by engaging in actions such as beginning staff meetings with a compassion practice, coaching colleagues who felt overwhelmed from the rapid transition to online teaching at the onset of the COVID-19 pandemic, and adjusting discipline policies to listen more to students’ perspectives; include them in solutions and next steps; and co-create a plan to attend to the unique needs of each student.

### Aspects of Co-design Inform Educators’ Thinking About Compassionate Schools

Educators attributed shifts in their interactions with students, students’ families, and colleagues to the contemplative practice, reflection, and joint inquiry they engaged in throughout the compassion training and co-design process. Because we introduced compassion training to educators as part of the co-design process, educators did not always distinguish between compassion training and the other components of the co-design process in their speech and writing.

#### Contemplative Practice Impacted Educators’ Interactions

Educators viewed the contemplative practices as foundational to improving their interactions and working toward more compassionate schools. Ruth shared that through participating in CCT, she did “some practice developing my own practice at home, talking with friends and colleagues about what I was learning.” Ruth explained that she did not have a regular contemplative practice prior to CCT: “I was starting from a baseline of not having a practice. So, you know, the only place I could go is up.” For Ruth, engaging in contemplative practice also encouraged awareness and reflection, particularly around the suffering of members of her school community. This, in turn, impacted her interactions as she reflected that the contemplative practices “just really did affect me and which carried over to my parenting, which carried over to my interactions with colleagues and my work with students, my understanding” (Interview). As the focus of the meetings shifted to the co-design work, she “really [tried] to dig into those aspects of working together to come up with a course that might really speak to educators and support what they needed moving forward” (Interview).

In their written reflections and interviews, educators referenced specific practices as influencing their ability to bring care and compassion to their interactions with members of their school community. Educators were introduced to the practices—such as breathing, intention setting, loving-kindness for others, and self-compassion—during the 8-week CCT, and they revisited these practices as part of the co-design process. Martin found the active compassion practice from CCT particularly useful in his school counseling work with students and their families, especially as he explained: “When you don’t see a change, to at least hold some of these students and families in my mind and do that practice. It seemed to provide some psychological relief. There can’t be any downside” (Interview). Other educators, such as Bridgette and Michael, spoke about using intention setting as well as breathing practices to settle their minds before and during challenging meetings with students, their families, and/or colleagues. Some educators, such as Ruth, shared that they “channeled” contemplative practice to be “more present with [students] as they navigated their challenges” (Reflection). As we explored previously, educators also discovered that the contemplative practices strengthened their capacity to care for themselves which, in turn, impacted their interactions with others in their school community. In their interviews and written reflections, educators reflected on the power of self-compassion practices.

#### Joint Inquiry and Reflection Impacted Educators’ Interactions

While contemplative practices served as a foundation for educators to skillfully care for themselves and members of their school community, it was joint inquiry and reflection that encouraged educators to envision possibilities for more equitable and just school environments. Martin experienced contemplative practices helpful in his role as an educator but recognized through joint inquiry that to be skillful in caring for the school community required attention to context, including the way systems and culture operates in his district. He shared,

I think there were some good conversations [in the co-design meetings] around the equity piece …To even sit down and contemplate these things and meditate and reflect, there’s a certain level of privilege that’s in there. I mean I appreciated that it was a part of a lot of conversations...As someone who really cares about that and works closely, especially with our Latino community, which tends to be in [my district] more underserved and families less resourced. (Interview)

Amara shared that the group discussions helped her to recognize the individual actions that she had with students in her classroom were examples of compassion, but that she could do “more on the structural level, how you structure the class” to create a more just and inclusive environment. It was through joint inquiry that she began to envision possibilities for doing so:

I need to create a different classroom culture...so I feel like the compassion work is more of, for me, a structural thing … that leads everybody to feeling [more comfortable] ... As a White educator, I’m looking and thinking, okay, how do I be more truly anti-racist? How I come across, and how I set up my class, and how I teach. (Interview)

Educators had opportunities to engage in joint inquiry during co-design meetings as the group analyzed sources of suffering in schools and used this analysis to inform the design of the digital compassion course. Jessica explained that during co-design meetings, “there was this space to kind of translate what we were dealing with in the classroom to how different that is through the [state], through the country, through the different age groups” (Interview). Educators shared that being able to discuss challenges to compassion and strategies for offering compassion supported them in taking compassionate action and in imagining their schools and schools around the country as more compassionate places.

Educators also wrote field notes, reflecting on opportunities for compassion, and engaged in joint inquiry around the field notes. Donna explained that the process of sharing and discussing field notes supported her to envision new discipline policies in her school that aligned with compassion. She said that several times during co-design,

People were talking about how we deal with discipline issues with compassion. And the whole idea of [a student’s] re-entry [into school] after something has happened, and helping students move beyond the incident and change that conversation. And I think about that with kids who I feel get labeled or because they’ve done one thing, then it’s hard to even break out of that. Whatever restorative justice or whatever the system is and take care of it. And then let’s move past it with a clean slate. So, I have thought about that in a number of cases, and specifically the field notes I wrote was a case of that in my teaching, where it was hard to move beyond because I think neither the student nor I had the closure we wanted. And so we worked on that. (Interview)

In Donna’s example, she and the student “moved beyond” a challenging classroom incident by talking and listening to one another over lunch the following day. In this example, Donna reflected that the process of writing and sharing field notes with her peers helped her to see her individual action with a student within a larger system of school discipline. Her vision of a compassionate school involved policies and practices that recognized the dignity of students, allowing for students to make mistakes and learn from their mistakes, and where the mistakes do not define them.

These educators’ reflections collectively speak to the complementarity of practice and joint inquiry and reflection as resources to develop a wider repertoire of caring responses to suffering in themselves, in their interactions with others, and in their schools. Practices helped to build inner resources to call upon in difficult interactions, many of which recur in schools, such as discipline and interactions with parents about the school performance of their children.

## Discussion

In this study, we explored the ways in which educators drew upon compassion training and the joint inquiry that occurred during co-design meetings to develop new insights about how to cultivate and offer compassion in their schools. The ten educators in this sample found numerous opportunities to approach social interactions through a lens of compassion. Educators viewed compassion as relevant and necessary in their professional lives. They often spoke about using self-compassion as a strategy throughout the day to support their own wellbeing and improve the quality of their relationships with others.

**Fig. 1:**
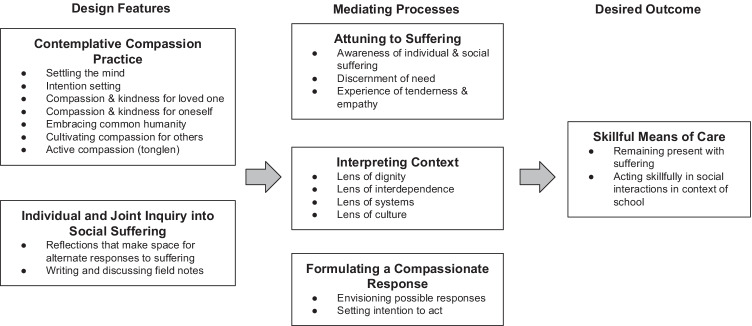
Conceptual model for developing educators’ skillful means of care

Our findings are the basis for a conceptual model for thinking about developing skillful means of care in educational settings articulated in Fig. 1. Skillful means of care can be cultivated through contemplative practice and inquiry into social forms of suffering that educators encounter in their interactions with students, families, and colleagues in schools. These two key design features, contemplative compassion practice and individual and joint inquiry into social suffering, support a number of observable interactions among educators and with design features, which we refer to as mediating processes because they are the means by which our design supports educators’ development (see Sandoval, 2013). These include helping educators attune to suffering, interpret their contexts in light of different lenses, and formulate a compassionate response. Specifically, activities supported educators in attuning to suffering by helping them become aware of individual and/or social suffering in their schools, discern a human need, and open to affective feelings of tenderness and empathy within the context of their schools. In addition, the activities provided lenses for interpreting their context, including through the lenses of dignity, interdependence, systems, and culture. Activities helped educators formulate a compassionate response to individual and social suffering by engaging them in envisioning possible responses they could take to alleviate suffering and in setting intentions to act with care. Educators cultivated skillful means of care through attuning to suffering, interpreting the context, and formulating a compassionate response. Together, these aspects of interaction with course activities contributed to the outcome of developing educators’ skillful means of care, which included remaining present in the face of suffering and acting skillfully in social interactions in the context of schools to alleviate the suffering.

Our findings align with previous research that highlights the benefits of compassion training for educators, particularly findings that point to educators’ capacity to employ compassion techniques in their interactions with others (Maratos et al, 2019; Taylor et al., 2016). We extend this research to argue that the adaptation of a general program on compassion training can benefit from attending to how to show compassion in the context of concrete interactions in schools—with self, others, and with organizational practices—and this, in turn, can support educators in developing skillful means of care. Based on our analysis, contemplative practice alone did not appear enough to support educators in expanding their vision to include compassionate school change. Through the co-design process, where educators had opportunities to cultivate compassion and engage in individual and joint inquiry and reflection together, they explored how they could offer compassion in schools to improve their interactions and work toward school change.

When engaging educators in developing skillful care in schools, our findings suggest that there is value in doing so through a participatory approach, such as co-design. The co-design process made it possible to weave together contemplative practice, reflection, and individual and joint inquiry in a way that supported educators’ development of skillful means of care. While we recognize that it is not always feasible to engage in a co-design process due to time, resources, or differing goals, this study highlights the potential contributions of including the elements of reflection and joint inquiry alongside contemplative practice when working with educators to create more compassionate schools.

This study suggests that contemplative compassion practice, reflection, and individual and joint inquiry together support educators in envisioning more compassionate environments and in engaging in caring actions in their schools. Further, our analyses provide insight into the components that supported this group of educators to cultivate and offer compassion in schools. Our analyses also suggest that educators’ skillful means of care can be cultivated through both contemplative practice and inquiry into social forms of suffering that educators encounter in schools. If we want educators to find joy in teaching and to care for their students and if we want students and their families to feel they belong in schools, we must find ways to support educators to envision new possibilities for their schools that begin to address the social suffering exacerbated by common policies and practices. Contemplative practice paired with individual and joint inquiry into social suffering in schools appears to be a promising step in this direction. While the contemplative practice, reflection, and individual and joint inquiry were key elements of the co-design process, a promising avenue for future research could investigate if other structures such as workshops or trainings for educators that feature contemplative practice, reflection, and individual and joint inquiry also support educators to enact compassionate change in schools.

In this study, educators recognized the ways that students suffered in schools and brought compassion to their interactions with students. This was especially true when working with students whose behaviors did not align with their own, school, or classroom expectations. Educators also noted that their interactions with colleagues benefited from compassion. Additionally, educators began to approach their interactions with students and students’ families through a lens of compassion in ways that honored their dignity. These findings align with previous studies on compassion training for educators that point to the promise of supporting educators’ wellbeing and promoting positive interactions with students and colleagues (Braun et al., 2020; Maratos et al., 2019; Taylor et al., 2016). As the co-design work deepened to develop a course focused on supporting educators to create more just and compassionate schools, the educators in this study also expanded their vision for what was possible in their own schools. Educators imagined possibilities for school change by revising long-standing routines and policies, such as grading and discipline, as a way to begin to attend to the suffering caused by schools.

While we presented each of these applications of compassion—interpersonal, personal, and community—separately in the findings, they did not occur in isolation (Rogoff, 1995). Rather, during a busy school day, educators might be faced with any number of opportunities for compassion in which they must decide how to skillfully care for members of their school community. Often, such as when teaching a class, educators care for students simultaneously. In fact, educators noted that the “breakneck pace of teaching” along with the focus on the future made it difficult to stay in the moment to care for themselves and their students. Educators found support and inspiration for offering compassion in their contexts from the compassion training and the co-design meetings. They attributed the shifts in their interactions with school community members to the contemplative practice and individual and joint inquiry into social suffering in schools. And, due in part to this inquiry, they expanded their capacity for imagining and working toward school change.

### Limitations and Future Research

Although this study contributes to our understanding of how educators cultivate compassion in their school settings and the components that support them to do so, there are several limitations. First, the majority of our analysis focused on the learning and reflections from ten educators in one district in the USA. The small-scale nature of this study constrains our ability to generalize findings across broader contexts. Furthermore, the majority of educators in this study identified as White and as female. There is a need for future studies examining the development of skillful means of care with more diverse and representative populations of educators, particularly as care is often viewed as highly gendered and invisible work (Acker, 1995; Collins, 2000; Puig de la Bellacasa, 2017). Additionally, this study coincided with the start of the global COVID-19 pandemic, presenting a unique and specific context for educators to practice compassion and for our own data collection. Nonetheless, we were impressed by educators’ commitment to the project and to the work of designing a compassion course for other educators, as they dedicated nearly 60 after-school hours and continued to participate in the midst of the uncertainty of the pandemic.

This group of educators, who applied for the yearlong project, brought to the project an orientation toward compassion and caring that might differ from a randomly selected sample of educators. Indeed, a topic of discussion in the co-design meetings was how to persuade colleagues who might need a compassion course but are also critical or skeptical of such an offering. Nonetheless, this study adds to the literature on mindfulness and compassion training programs for educators and suggests educators see compassion as a necessary and integral component of their work in schools and that they can be supported to strengthen their capacities. Future research should identify entry points for educators who do not immediately recognize the value of compassion and the importance of caring for their school communities.

We relied upon educators’ self-reports regarding how they applied compassion in their interactions at schools, including semi-structured interviews. While semi-structured interviews allow for the interviewer to ask follow-up and clarifying questions, such interviews also have the potential to influence an interviewee’s response based upon the questions asked. An important next step in the research is to understand what it looks like to skillfully enact care and compassion in schools. Developing instruments, such as observation protocols, and methodologies for identifying acts of care and compassion that often remain invisible in schools would help us to document these actions. An additional line of research focused on critical caring would be to collect data on students’ perspectives to investigate if the acts of care and compassion educators report are perceived by students as skillful. Drawing inspiration from Garza’s (2009) research, which used open-ended student questionnaires to identify similarities and differences in Latinx and White students’ perceptions of teacher caring, future research should attend to the ways acts of compassion are contextualized and what skillful means of care looks like in different school communities.

As interventions to support mindfulness and compassion continue to expand to support specific groups of professionals, an ongoing focus of inquiry into how such interventions support application in their professional setting is critical. In this study, elaborating on the concept of skillful means to consider what skillful caring and compassionate action looks like in the context of schools illuminated the complementary roles played by contemplative practice and joint inquiry. Joint inquiry that included engagement with literature related to caring in schools, as well as opportunities for educators to write about and discuss their interactions, helped them to identify new responses to suffering in their workplace environment. Perhaps because of their shared orientation and their familiarity with the dynamics common to school communities, educators learned much from one another in this context.

A key aim of compassion training is to help people respond to suffering differently, from an intention to help alleviate suffering. This study affirms the potential of such training, when coupled with joint inquiry, in helping educators to envision different ways to respond with compassion to the suffering of students, in difficult interactions with colleagues and parents, and to their own reactions to difficulty. At the same time, throughout the study, educators identified many more opportunities to practice compassion than they were able to act upon in the moment, as well as larger structures in their school related to discipline and grades that were likely to continue to create suffering for others. This study thus represents a promising beginning to a line of research that invites broader consideration of how compassion might be developed in schools as systems by simultaneously supporting the individual development of educators and joint inquiry and action to reduce sources of suffering in schools today.
